# Comparison of Epiretinal Membrane Detection Rates Between Optos^®^ and Clarus^™^ Ultra-Widefield Fundus Imaging Systems

**DOI:** 10.3390/jcm15020883

**Published:** 2026-01-21

**Authors:** Satoshi Kuwayama, Yoshio Hirano, Arisa Shibata, Hiroaki Sugiyama, Nariko Soga, Kihei Yoshida, Takaaki Yuguchi, Ryo Kurobe, Akiyo Tsukada, Shuntaro Ogura, Hiroya Hashimoto, Tsutomu Yasukawa

**Affiliations:** 1 Department of Ophthalmology & Visual Science, Nagoya City University Graduate School of Medical Sciences, 1-Kawasumi, Mizuho-cho, Mizuho-ku, Nagoya 467-0001, Aichi, Japan; manga.anime.takkyuu@gmail.com (S.K.); marron11141231@gmail.com (H.S.);; 2 Department of Ophthalmology, Ogaki Tokushukai Hospital, Ogaki 503-0015, Gifu, Japan; 3 Clinical Research Management Center, Nagoya City University Hospital, Nagoya 467-0001, Aichi, Japan; hiroya.hashimoto@nnh.go.jp

**Keywords:** ultra-widefield retinal imaging systems, epiretinal membrane, fundus photography, Optos^®^, Clarus^™^

## Abstract

**Background:** Ultra-widefield (UWF) images are frequently used for fundus examinations during medical screening. Optos^®^ generates pseudo-color images using only red and green lasers, which may reduce the visibility of retinal interface lesions. In contrast, Clarus™ incorporates blue light, suggesting potential superiority in epiretinal membrane (ERM) detection. **Methods:** This retrospective study included 233 patients (408 eyes; 816 UWF images per device) who underwent simultaneous Optos^®^ and Clarus™ imaging plus optical coherence tomography (OCT) at our institution from March to April 2019. Ten blinded ophthalmologists assessed only the UWF images for ERM presence or absence. Diagnosis was confirmed by fundus examination and OCT. McNemar’s test compared detection accuracy. **Results:** Clarus™ consistently outperformed Optos^®^, with superior sensitivity [median 49% (range 42–70) vs. 14% (4–47); *p* = 0.002], correct judgment rate [85% (82–90) vs. 78% (44–88); *p* = 0.010], and lower unassessed rate [6% (2–13) vs. 13% (3–52); *p* = 0.002]. This superiority held across ERM stages, lens status, and ophthalmologist experience levels. **Conclusions:** This study demonstrated that Clarus™ significantly outperformed Optos^®^ in ERM detection accuracy. These results suggest that true-color UWF systems like Clarus™ may be more useful for macular screening in routine practice and health examinations.

## 1. Introduction

A fundus camera is typically used for fundus examination during medical check-ups [1]. In recent years, an ultra-widefield (UWF) fundus camera was developed, which is particularly useful in routine clinical practice for the detection of abnormalities in peripheral regions [2,3]. This UWF technology is now being used in human medical check-ups. One widely used UWF imaging system is the Optos^®^ camera (Optos PLC, Dunfermline, Scotland, UK), which captures a 200-degree field of the fundus in a single image, without requiring pupil dilation. However, the resulting images are pseudo-color images, generated from red and green lasers (lacking a blue channel), not true-color images.

In contrast, Zeiss Clarus^™^ (Carl Zeiss Meditec, Jena, Germany) is a newer scanning laser ophthalmoscope that uses red, green, and blue light-emitting diode lasers to produce true-color images and can capture a 133-degree view in a single shot without pupil dilation [3,4]. Although Clarus^™^ can also produce a 200-degree image like Optos^®^, it requires two shots to create a composite image. The advantage of Clarus^™^ over Optos^®^ is that it reduces artifacts from lashes and eyelids [5]. In our previous study, Optos^®^ was found to be superior to a conventional fundus camera with a view angle of approximately 45° in detecting peripheral retinal lesions such as detachment and breaks, but it was less effective in detecting macular diseases such as epiretinal membrane (ERM) (unpublished data). While some studies have compared Optos^®^ and Clarus^™^ in diseases such as age-related macular degeneration (AMD) [5] and diabetic retinopathy (DR) [4,6], none have investigated their ability to detect ERM. Therefore, this study aimed to compare the diagnostic accuracy of ERM detection between the Optos^®^ and Clarus^™^ UWF imaging systems by conducting fundus imaging on a group of patients.

## 2. Methods

### 2.1. Patients

This study was retrospective and observational, and was approved by the Institutional Review Board of Nagoya City University Graduate School of Medicine (No. 60-22-0021). Because the study was retrospective, written informed consent was not obtained from all patients. Instead, a website was created to provide information about the purpose of the study that the patients could read. The research methods and analyses adhered to the principles of the Declaration of Helsinki. The study included 233 patients who visited Nagoya City University Hospital between March 2019 and April 2019, comprising 408 eyes and 816 ultra-widefield fundus images (408 per device). Patients underwent fundus photography of the same eye using two different cameras, Optos^®^ (Optos California, Optos PLC, Dunfermline, Scotland, UK) and Clarus^™^ 500 (Carl Zeiss Meditec, Jena, Germany), as well as optical coherence tomography (OCT) imaging. The patients also underwent ophthalmologic examinations, including measurement of best-corrected visual acuity, slit-lamp examination, indirect ophthalmoscopy, fundus photography, and OCT (Cirrus HD-OCT 6000; Carl Zeiss Meditec, Jena, Germany) imaging. The inclusion criteria for the study were patients who underwent fundus photography of the same eye with both Optos^®^ and Clarus^™^, while the exclusion criteria were patients who did not agree to participate in the study and/or those with poor imaging.

### 2.2. Assessments

A total of 816 Optos^®^ and Clarus^™^ images of 408 eyes were assessed by ten ophthalmologists, including four 1st-year ophthalmologists (A.S., H.S., N.S., and K.Y.) and six ophthalmologists with ≥2 years of experience [S.K., Y.H., A.S., H.S., N.S., K.Y., T.Y. (Takaaki Yuguchi), R.K., A.T., and S.O.]; mean 9 years of experience, range 3–21 years.

The images were divided into three categories: eyes with ERM, eyes without ERM, or classified as unassessed. Fundus examination and/or OCT imaging were used to correctly determine the presence or absence of ERM. Furthermore, stage classification of ERM was performed according to a previous report [7], and the detection accuracy of ERM for each stage was compared between the two models. The sensitivity (positive predictive value), specificity (negative predictive value), false positives (wrongly judged as present), false negatives (wrongly judged as absent), correct judges (correctly judged as present or absent ERM), incorrect judges (wrongly judged as present or absent ERM), and unassessed were calculated for each reader, and the means for each value were also calculated.

### 2.3. Statistics

Statistical analysis was performed using Easy R software Ver. 1.70 (Jichi Medical University Saitama Medical Center, Saitama, Japan), which is a graphical user interface for R Ver. 4.20 (The R Foundation for Statistical Computing, Vienna, Austria). More precisely, it is a modified version of R commander designed to add statistical functions frequently used in biostatistics [8]. *p* < 0.05 was set as significantly different. All values are expressed as the mean ± standard deviation or median with range. The medians of sensitivity, specificity, false positives, false negatives, correct judges, wrong judges, and unassessed were compared between Optos^®^ and Clarus^™^ images using Wilcoxon’s signed rank test after calculating the results on each imaging system for each reader. In addition, the ERM detection accuracy was compared between the two models by McNemar’s analysis, considering the presence or absence of ERM, the cause of ERM, lens status, and years of ophthalmologist experience.

## 3. Results

Table 1 shows the baseline characteristics of the patients in this study. A total of 233 patients, consisting of 119 men and 114 women, with 408 eyes (208 men and 200 women) and a mean age of 65 ± 14 years, were included. Of these, 53 eyes had ERM, of which stages 1, 2, 3, and 4 were present in 19, 22, 10, and 2 eyes, respectively, whereas 355 eyes did not have ERM. Among the eyes without ERM, 83 had no ocular diseases, 83 had DR, 52 had AMD, 40 had retinal vein occlusion, and 97 had other eye diseases.

The study assessed the sensitivity, specificity, false positives, false negatives, and accuracy of ERM detection by ten ophthalmologists, including four ophthalmologists in their first year of ophthalmology experience. Sensitivity (positive predictive value) was defined as correct identification of ERM, and specificity (negative predictive value) was defined as accurate identification of the absence of ERM. The median (with range) sensitivity in the Clarus^™^ group [49 (42–70)%] was higher than that in the Optos^®^ group [14 (4–47)%] (*p* = 0.002) (Table 2), and the median number of correct judges (correctly judged as present or absent) was also higher in the Clarus^™^ group [85 (82–90)%] than in the Optos^®^ group [78 (44–88)%] (*p* = 0.010) (Table 2), indicating better ERM detection accuracy for Clarus^™^. There was no significant difference in the median specificity between the two groups (88 (48–97)% in the Optos^®^ group and 89 (87–96)% in the Clarus^™^ group; *p* = 0.186) (Table 2). The median false positives were higher in the Clarus^™^ group (*p* = 0.002), while both mean false negatives and unassessed were higher for Optos^®^ (*p* = 0.012 and *p* = 0.002, respectively) than for Clarus^™^ (Table 2). Subgroup analysis was performed for each rater owing to variations in experience among the 10 graders (Supplementary Table S1). A similar pattern was identified in the subgroup analysis for sensitivity, false positives, false negatives, correct judgments, and unassessed cases, highlighting significant disparities between the two models, as indicated in Table 2.

McNemar’s analysis was performed to assess the accuracy of detecting the presence or absence of ERM. Clarus^™^ had a significantly higher detection accuracy than Optos^®^ in cases with and without ERM (Table 3). A comparison of the detection accuracies for each ERM stage of the two models is presented in Table 4. In all stages, Clarus^™^ had a significantly higher detection accuracy for ERM than Optos^®^. Clarus™ demonstrated superior detection rates across all stages: Stage 1 (approximately 27% vs. 5%), Stage 2 (69% vs. 25%), Stage 3 (54% vs. 34%), and Stage 4 (95% vs. 50%), all with *p* < 0.001 (McNemar’s test). Table 5 shows the differences in the detection rates of the ERM between the two models based on the lens status. In phakic eyes, Clarus^™^ had a significantly higher percentage of correct judgments than Optos^®^, regardless of the presence or absence of ERM (*p* < 0.001). In pseudophakic eyes, the rate of correct judgment was significantly higher for Clarus^™^ in eyes with ERM, but there was no significant difference in eyes without ERM. Neither group showed any correct judgment in aphakic eyes with ERM, and the *p*-value could not be measured. In aphakic eyes without ERM, there was a slightly significant difference, with Optos^®^ showing a higher accuracy.

In terms of the percentage of correct judges for ERM detection based on years of ophthalmologist experience, both first-year and more experienced ophthalmologists had significantly higher rates of correct judges when using Clarus^™^, regardless of the presence or absence of ERM (Table 6).

## 4. Representative Cases

### 4.1. Case 1

**Figure 1:**
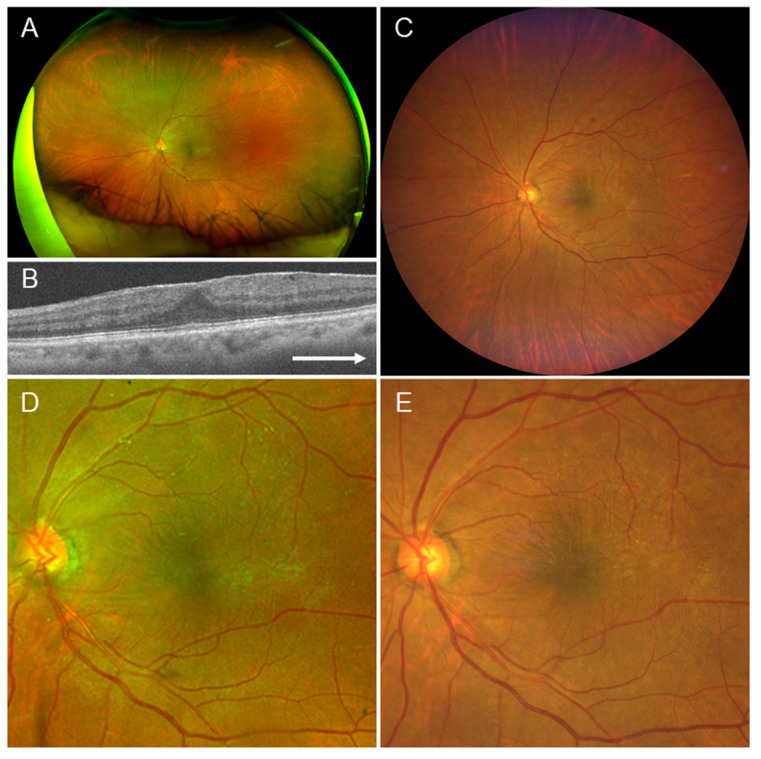
Findings in a 67-year-old female with mild cataract, non-proliferative diabetic retinopathy, and secondary epiretinal membrane (ERM) in the left eye. This is a case in which all 10 readers judged the presence of ERM in both the Clarus^™^ and Optos^®^ image. (**A**) Optos^®^ image with a 200-degree field. (**B**) Optical coherence tomography horizontal image, showing ERM. (**C**) Clarus^™^ image with a 130-degree field. (**D**) Optos^®^ image with a 50-degree field, showing reflexes on the retinal surface and retinal wrinkling, indicating the presence of ERM. (**E**) Clarus^™^ image with a 50-degree field, also clearly showing ERM. Arrow: The arrow means that it is a horizontal image.

A 67-year-old female. The patient had mild cataracts, mild non-proliferative DR, and secondary ERM (Figure 1). In this case, ERM, reflection on the retinal surface, and wrinkling of the retina were clearly visible in both the Optos^®^ and Clarus^™^ images, and all 10 readers confirmed the presence of ERM in both images (Table 7).

### 4.2. Case 2

**Figure 2:**
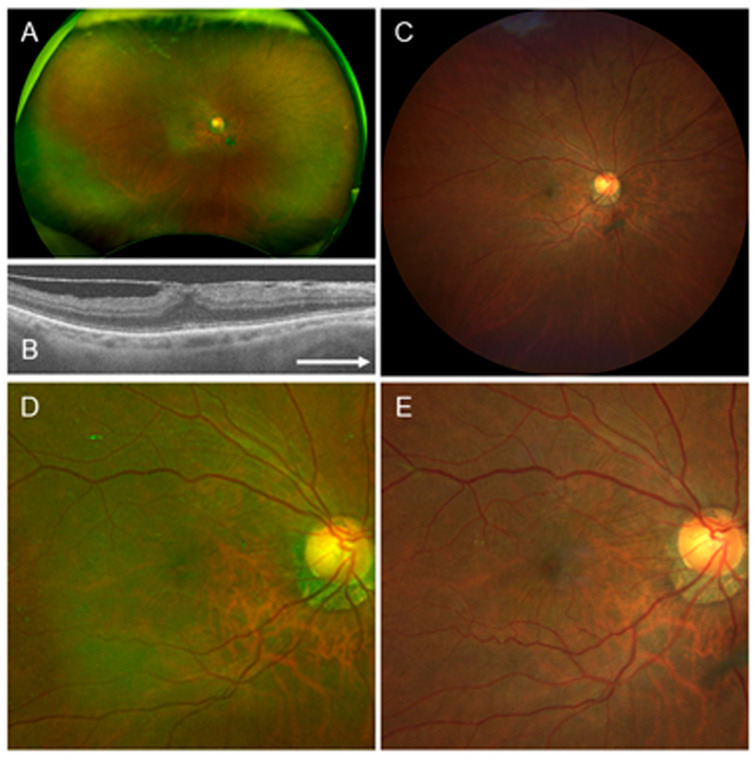
Findings in a 71-year-old female with mild cataract and idiopathic epiretinal membrane (ERM) in the right eye. This is a case in which the rate of ERM diagnosis was much higher on the Clarus^™^ image than the Optos^®^ image. (**A**) Optos^®^ image with a 200-degree field. (**B**) Optical coherence tomography horizontal image, showing ERM. (**C**) Clarus^™^ image with a 130-degree field. (**D**) Optos^®^ image with a 50-degree field, showing a slightly blurred retinal image that might be misdiagnosed as the absence of ERM. (**E**) Clarus^™^ image with a 50-degree field, showing ERM and distinct retinal wrinkling. Arrow: The arrow means that it is a horizontal image.

A 71-year-old female. The patient had a mild cataract and idiopathic ERM in the right eye (Figure 2). In this case, retinal wrinkling was clearly visible in the Clarus^™^ image, while the Optos^®^ image was blurredly visible and ERM was not evident. Therefore, while all ten readers judged the presence of ERM in the Clarus^™^ image, only one reader judged the presence of ERM in the Optos^®^ image (Table 7).

### 4.3. Case 3

**Figure 3:**
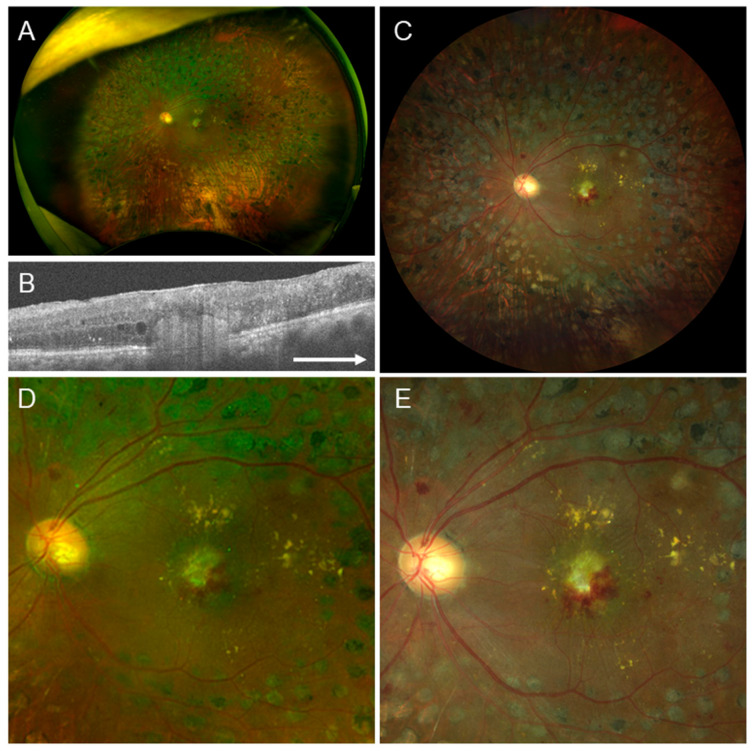
Findings in a 68-year-old male, whose left eye had pseudophakia, non-proliferative diabetic retinopathy, exudative age-related macular degeneration, and secondary epiretinal membrane (ERM). This is another case in which the rate of ERM diagnosis was much higher on the Clarus^™^ image than the Optos^®^ image. (**A**) Optos^®^ image with a 200-degree field. (**B**) Optical coherence tomography horizontal image, showing ERM and hyperreflective material indicating active fibrosis and subretinal hemorrhages. (**C**) Clarus^™^ image with a 130-degree field. (**D**) Optos^®^ image with a 50-degree field, clearly showing subretinal hemorrhages and subretinal fibrosis at the macula, but ERM is not evident. (**E**) Clarus^™^ image with a 50-degree field, showing retinal wrinkling indicating ERM, in addition to the hemorrhages and fibrosis. Arrow: The arrow means that it is a horizontal image.

A 68-year-old male. The left eye had pseudophakia, moderate non-proliferative DR, exudative AMD, and secondary ERM (Figure 3). In this case, retinal wrinkling was visible in the Clarus^™^ image, and 9 of 10 readers confirmed the presence of ERM. However, in the Optos^®^ image, it was difficult to observe retinal wrinkling, and all 10 readers reported the absence of ERM (Table 7).

### 4.4. Case 4

**Figure 4:**
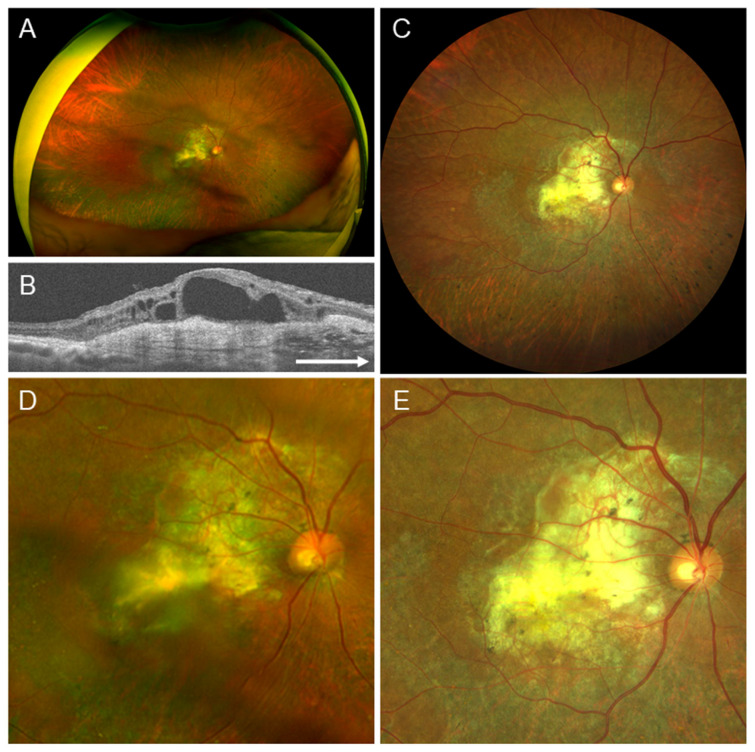
Findings in a 77-year-old male, whose right eye had pseudophakia, exudative age-related macular degeneration, and secondary epiretinal membrane (ERM). This case represents a low diagnostic rate of ERM in both the Optos^®^ and Clarus^™^ image. (**A**) Optos^®^ image with a 200-degree field is entirely blurred. (**B**) Optical coherence tomography horizontal image, showing thin ERM, cystoid macular edema, and subretinal fibrosis. (**C**) Clarus^™^ image with a 130-degree field. (**D**) Optos^®^ image with a 50-degree field, which is blurred particularly in the inferior area, showing subretinal fibrosis, but ERM is not evident. (**E**) Clarus™ image with a 50-degree field, which is consistently clear, with subretinal fibrosis being prominently visible. However, the presence of ERM is not readily apparent. Arrow: The arrow means that it is a horizontal image.

A 77-year-old male. The right eye had pseudophakia with advanced AMD and secondary ERM (Figure 4). In this case, the severity of accompanying findings, such as subretinal fibrosis and cystoid macular edema, made it difficult to distinguish the presence of ERM in both images (Table 7).

## 5. Discussion

We conducted this study to compare the rate of ERM detection between the Optos^®^ and Clarus^™^ UWF imaging systems. In this study, Clarus^™^ demonstrated significantly higher rates of sensitivity (positive predictive value), correct judgment (correctly judged as present or absent ERM), and overall accuracy compared to Optos^®^ (Table 2). Optos^®^ produces pseudo-color images generated from red and green lasers (lacking a blue channel), whereas Clarus™ uses red, green, and blue light-emitting diode lasers to produce true-color images that closely resemble the actual color of the retina [3]. Green laser light reflects the retinal pigment epithelium, while red laser light reflects on the choroid, resulting in no reflection at the retinal surface [2]. Therefore, it may be difficult to depict the ERM, which is located on the retinal surface. In addition, Clarus^™^ has a higher resolution (7 µm) than Optos^®^ (14 µm), which allows for more accurate determination of ERMs [5,9]. Taken together, it is assumed that Clarus^™^ had higher detection rates for ERM than Optos^®^. The blue channel’s role is supported by studies showing improved contrast for retinal interface lesions via short-wavelength light reflectance, which particularly enhances the visualization of surface abnormalities such as ERM and retinal nerve fiber layer defects [10,11]. Previous reports have compared Optos^®^ and Clarus^™^ in detecting diseases such as DR and AMD [4,5,6]. Clarus^™^ was found to have the highest sensitivity for detecting neovascularization in AMD compared to Optos^®^ and digital fundus cameras [5]. Additionally, reports indicate that Clarus^™^ is superior to Optos^®^ in detecting lesions in the central macula for DR findings such as microaneurysms and retinal hemorrhages [6]. The superiority of Clarus™ was consistent across all ERM stages, with particularly pronounced differences in early stages (Stages 1 and 2), where subtle surface membranes are more challenging to detect with pseudo-color imaging. This finding aligns with Govetto’s optical coherence tomography-based staging scheme, in which early-stage ERM is characterized by thin, transparent membranes that benefit from the enhanced contrast provided by true-color imaging [7]. In contrast, Clarus^™^ had a higher rate of false positives (wrongly judged as present) (Table 2), possibly due to mistaking physiological macular reflexes for ERMs because of the higher resolution, but there were few cases available for the evaluation of false positives. In contrast, Optos^®^ had higher rates of false negatives (wrongly judged as absent) and unassessed (Table 2), which may be due to resolution issues or the effects of artifacts, such as eyelashes [5,9].

According to McNemar’s analysis, Clarus^™^ demonstrated superior accuracy compared to Optos^®^ in detecting ERMs across all categories, including the presence or absence of ERM (Table 3), ERM staging (Table 4), lens status (Table 5), and years of ophthalmologist experience (Table 6), indicating that it is effective in detecting ERM. Regarding secondary ERM, as demonstrated in Figure 3, when the findings of primary diseases were mild, Clarus^™^ had a higher rate of ERM detection. However, as shown in Figure 4, when the findings of the primary diseases were severe, both Clarus^™^ and Optos^®^ had lower rates of ERM detection.

Regarding lens status, Clarus^™^ was also superior in phakic patients, suggesting that it was less affected by cataract artifacts (Table 5A,B). In patients with pseudophakic eyes and ERM, the diagnostic rate of ERM was similar to that of Clarus^™^ than Optos^®^ (Table 5A). In contrast, there was no significant difference between Optos^®^ and Clarus^™^ in the absence of ERM (Table 5B), but the reason for this was unclear. In eyes with aphakia and ERM, all readers judged the absence of ERM or unassessed, possibly due to the difficulty in distinguishing ERM in two cases of severe myopia. For aphakic patients without ERM, Optos^®^ showed slightly better results (Table 5B); however, further research is needed because of the limited number of cases.

Regarding the years of ophthalmologist experience, ERM detection rates were better for Clarus^™^ than for Optos^®^, regardless of the presence or absence of ERM (Table 6A,B). Thus, although Optos^®^ is inferior to Clarus^™^ in detecting ERM, one advantage is its wider range of view, which enables a higher detection rate of peripheral lesions [6,9,12] and allows for obtaining more information on the retina than Clarus^™^, as reported in previous studies [12]. Therefore, it may be preferable to analyze the characteristics of both models and utilize each model based on the specific lesion being targeted.

Based on these findings, we found that Clarus^™^ generally exhibited a superior rate of ERM detection compared with Optos^®^. We believe that Clarus^™^ could be more valuable when assessing ERM detection in medical check-up screening.

The current study has several limitations that must be considered. Firstly, it was a retrospective study that only took place in one institution and only included Japanese patients. Additionally, background diseases such as diabetic retinopathy, age-related macular degeneration, and retinal vein occlusion may potentially interfere with ERM detection due to masking effects from hemorrhages or exudates. Although OCT confirmation mitigated diagnostic bias, subgroup analysis by underlying disease was not performed due to the retrospective design and sample constraints, representing a further limitation [13]. Additionally, the analysis was conducted using single-shot images obtained from both Optos^®^ and Clarus^™^, resulting in different fields of view. However, since ERM is primarily a macular lesion, detection relied on zoomed evaluation of the central field, minimizing peripheral influence. No significant differences were observed between right and left eyes, consistent with retinal symmetry. Moreover, this study did not establish specific criteria for important factors such as the size, extent, or thickness of the ERM, which may lead to differences in interpretation between thin and thick ERM cases. Reader- and analysis-related limitations include the absence of dedicated training protocols or formal inter-observer agreement assessments, potentially contributing to variability reflected in the wide metric ranges. Additionally, initial McNemar tables combined ‘no ERM’ and ‘unassessed’ categories (now separated in revisions), and small subgroups such as aphakic eyes limited robust interpretation, with minor counting discrepancies in pseudophakic data corrected.

Despite these limitations, the study found that Clarus^™^ was superior to Optos^®^ in detecting ERM. Furthermore, this study was conducted using 2019 data with the Optos California device primarily in pseudo-color (red/green) mode. Subsequent advancements in Optos technology, including the introduction of ultra-widefield true-color RGB imaging modality in 2023 [14] (incorporating a blue laser for enhanced visualization of superficial retinal structures such as epiretinal membranes), may reduce the differences observed in ERM detection between the two systems.

In conclusion, future studies should aim to address these limitations by conducting prospective multicenter studies involving a large number of cases. This would provide more reliable and comprehensive data on the effectiveness of these devices for detecting ERM.

## Figures and Tables

**Table 1:** Patient baseline characteristics.

Number of Patients	**233**
Number of eyes	408
Age (mean ± SD), years	65 ± 14 (20–87)
Gender (male/female), *n*	119/114
Lens status (phakia/pseudophakia/aphakia), *n*	271/131/6
Eyes with ERM, *n*	53
Stage 1 ERM, *n*	19
Stage 2 ERM, *n*	22
Stage 3 ERM, *n*	10
Stage 4 ERM, *n*	2
Idiopathic ERM, *n*	30
Secondary ERM, *n*	23
Eyes without ERM, *n*	355
Without any ocular diseases, *n*	83
DR, *n*	83
AMD, *n*	52
RVO, *n*	40
Others, *n*	97

SD, standard deviation. n, number. ERM, epiretinal membrane. DR, diabetic retinopathy. AMD, age-related macular degeneration. RVO, retinal vein occlusion. Classification of idiopathic vs. secondary ERM was based on the absence or presence of underlying retinal diseases (e.g., RVO or diabetic retinopathy). Comorbidities were not excluded, as ERM diagnosis was confirmed by OCT.

**Table 2:** Comparison of ERM detection accuracy between two devices by 10 readers.

Rate (%)	Optos^®^	Clarus^TM^	*p* Value (Wilcoxon’s Signed Rank Test)
Sensitivity	14 (4–47)	49 (42–70)	0.002 **
False positives (wrongly judged as present)	1 (0–5)	3 (1–11)	0.002 **
Specificity	88 (48–97)	89 (87–96)	0.186
False negatives (wrongly judged as absent)	53 (23–85)	40 (28–49)	0.012 *
Correct judges (rightly judged as present or absent)	78 (44–88)	85 (82–90)	0.010 *
Wrong judges (wrongly judged as present or absent)	9 (4–12)	8 (6–13)	0.846
Unassessed	13 (3–52)	6 (2–13)	0.002 **

* *p* < 0.05; ** *p* < 0.01 (Wilcoxon’s signed rank test). ERM, epiretinal membrane. Data are presented as median (range).

**Table 3:** Comparison of detection accuracy with or without ERM between two devices.

**Eyes with ERM ^(1)^** **(*n* = 53 × 10 Readers)**		**Clarus^TM^**		
		**Judged as Having ERM, *n***	**Judged as Having No ERM or Classified as Unassessed, *n***	**Total, *n***
**Optos^®^**	Judged as Having ERM, *n*	97	12	109
	Judged as Having No ERM or Classified as Unassessed, *n*	178	243	421
	Total, *n*	275	255	530
**Eyes without ERM ^(2)^** **(*n* = 355 × 10 Readers)**		**Judged as Having No ERM, *n***	**Judged as Having ERM or Classified as Unassessed, *n***	**Total, *n***
**Optos^®^**	Judged as Having no ERM, *n*	2730	212	2942
	Judged as Having ERM or Classified as Unassessed, *n*	474	134	608
	Total, *n*	3204	346	3550

ERM, epiretinal membrane. N, number. ^(1,2)^ *p* < 0.001 (McNemar’s test). The category ‘judged as having no ERM or classified as unassessed’ combines ‘no ERM’ and ‘unassessed’ judgments for binary McNemar analysis. Unassessed judgments were more frequent with Optos^®^ than Clarus™ (median per reader: 13 vs. 6; see Table 2), contributing to its lower detection accuracy. Exact distribution within the combined category is not separated into the original analysis, but the higher unassessed rate with Optos^®^ is acknowledged.

**Table 4:** Comparison of ERM detection accuracy by ERM stage.

**ERM Stage 1 ^(1)^** **(*n* = 19 × 10 Readers)**		**Clarus^TM^**		
		**Judged as Having ERM, *n***	**Judged as Having No ERM or Classified as Unassessed, *n***	**Total, *n***
**Optos^®^**	Judged as Having ERM, *n*	10	1	11
	Judged as Having No ERM or Classified as Unassessed, *n*	41	138	179
	Total, *n*	51	139	190
**ERM Stage 2 ^(2)^** **(*n* = 22 × 10 Readers)**		**Judged as Having ERM, *n***	**Judged as Having No ERM or Classified as Unassessed, *n***	**Total, *n***
**Optos^®^**	Judged as Having ERM, *n*	53	1	54
	Judged as Having No ERM or Classified as Unassessed, *n*	98	68	166
	Total, *n*	151	69	220
**ERM Stage 3 ^(3)^** **(*n* = 10 × 10 Readers)**		**Judged as Having ERM, *n***	**Judged as Having No ERM or Classified as Unassessed, *n***	**Total, *n***
**Optos^®^**	Judged as Having ERM, *n*	24	10	34
	Judged as Having No ERM or Classified as Unassessed, *n*	30	36	66
	Total, *n*	54	46	100
**ERM Stage 4 ^(4)^** **(*n* = 2 × 10 Readers)**		**Judged as Having ERM, *n***	**Judged as Having No ERM or Classified as Unassessed, *n***	**Total, *n***
**Optos^®^**	Judged as Having ERM, *n*	10	0	10
	Judged as Having No ERM or Classified as Unassessed, *n*	9	1	10
	Total, *n*	19	1	20

ERM, epiretinal membrane. N, number. ^(1–4)^ *p* < 0.001 (McNemar’s test). The category ‘judged as having no ERM or classified as unassessed’ combines ‘no ERM’ and ‘unassessed’ judgments for binary McNemar analysis. Unassessed judgments were more frequent with Optos^®^ than Clarus™ (median per reader: 13 vs. 6; see Table 2), contributing to its lower detection accuracy. Exact distribution within the combined category is not separated into the original analysis, but the higher unassessed rate with Optos^®^ is acknowledged.

**Table 5:** Comparison of ERM detection accuracy by lens status.

**(A) Case with ERM.**				
**Phakia ^(1)^** **(*n* = 27 × 10 Readers)**		**Clarus^TM^**		
		**Judged as Having ERM, *n***	**Judged as Having No ERM or Classified as Unassessed, *n***	**Total, *n***
**Optos^®^**	Judged as Having ERM, *n*	60	5	65
	Judged as Having No ERM or Classified as Unassessed, *n*	102	103	205
	Total, *n*	162	108	270
**Pseudophakia ^(2)^** **(*n* = 26 × 10 Readers)**		**Judged as Having ERM, *n***	**Judged as Having No ERM or Classified as Unassessed, *n***	**Total, *n***
**Optos^®^**	Judged as Having ERM, *n*	37	7	44
	Judged as Having No ERM or Classified as Unassessed, *n*	76	120	196
	Total, *n*	113	127	240
**Aphakia ^(3)^** **(*n* = 2 × 10 Readers)**		**Judged as Having ERM, *n***	**Judged as Having No ERM or Classified as Unassessed, *n***	**Total, *n***
**Optos^®^**	Judged as Having ERM, *n*	0	0	0
	Judged as Having No ERM or Classified as Unassessed, *n*	0	20	20
	Total, *n*	0	20	20
**(B) Case Without ERM.**				
**Phakia ^(4)^** **(*n* = 244 × 10 Readers)**		**Judged as Having No ERM, *n***	**Judged as Having ERM or Classified as Unassessed, *n***	**Total, *n***
**Optos^®^**	Judged as Having No ERM, *n*	1968	95	2063
	Judged as Having ERM or Classified as Unassessed, *n*	337	40	377
	Total, *n*	2305	135	2440
**Pseudophakia ^(5)^** **(*n* = 107 × 10 Readers)**		**Judged as Having No ERM, *n***	**Judged as Having ERM or Classified as Unassessed, *n***	**Total, *n***
**Optos^®^**	Judged as Having No ERM, *n*	737	110	847
	Judged as Having ERM or Classified as Unassessed, *n*	137	86	223
	Total, *n*	874	196	1070
**Aphakia ^(6)^** **(*n* = 4 × 10 Readers)**		**Judged as Having No ERM, *n***	**Judged as Having ERM or Classified as Unassessed, *n***	**Total, *n***
**Optos^®^**	Judged as Having No ERM, *n*	24	8	32
	Judged as Having ERM or Classified as Unassessed, *n*	1	7	8
	Total, *n*	25	15	40

ERM, epiretinal membrane. N, number. ^(1,2,4)^ *p* < 0.001, ^(3)^ P: unmeasurable, ^(5)^ *p* = 0.098, ^(6)^ *p* = 0.045 (McNemar’s test). The category ‘judged as having no ERM or classified as unassessed’ combines ‘no ERM’ and ‘unassessed’ judgments for binary McNemar analysis. Unassessed judgments were more frequent with Optos^®^ than Clarus™ (median per reader: 13 vs. 6; see Table 2), contributing to its lower detection accuracy. Exact distribution within the combined category is not separated in the original analysis, but the higher unassessed rate with Optos^®^ is acknowledged.

**Table 6:** Comparison of ERM detection accuracy by years of ophthalmologist’s experience.

**(A) Case with ERM.**				
**Ophthalmologists with 1 Year of Experience ^(1)^** **(*n* = 53 × 4 Readers)**		**Clarus^TM^**		
		**Judged as Having ERM, *n***	**Judged as Having No ERM or Classified as Unassessed, *n***	**Total, *n***
**Optos^®^**	Judged as Having ERM, *n*	22	1	23
	Judged as Having No ERM or Classified as Unassessed, *n*	82	107	189
	Total, *n*	104	108	212
**Ophthalmologists with at Least 2 Years of Experience ^(2)^** **(*n* = 53 × 6 Readers)**		**Judged as Having ERM, *n***	**Judged as Having No ERM or Classified as Unassessed, *n***	**Total, *n***
**Optos^®^**	Judged as Having ERM, *n*	75	11	86
	Judged as Having No ERM or Classified as Unassessed, *n*	96	136	232
	Total, *n*	171	147	318
**(B) Case Without ERM.**				
**Ophthalmologists with 1 Year of Experience ^(3)^** **(*n* = 355 × 4 Readers)**		**Judged as Having No ERM, *n***	**Judged as Having ERM or Classified as Unassessed, *n***	**Total, *n***
**Optos^®^**	Judged as Having No ERM, *n*	1151	68	1219
	Judged as Having ERM or Classified as Unassessed, *n*	166	35	201
	Total, *n*	1317	103	1420
**Ophthalmologists with at Least 2 Years of Experience ^(4)^** **(*n* = 355 × 6 Readers)**		**Judged as Having No ERM, *n***	**Judged as Having ERM or Classified as Unassessed, *n***	**Total, *n***
**Optos^®^**	Judged as Having No ERM, *n*	1579	144	1723
	Judged as Having ERM or Classified as Unassessed, *n*	308	99	407
	Total, *n*	1887	243	2130

ERM, epiretinal membrane. N, number. ^(1–4)^ *p* < 0.001 (McNemar’s test). The category ‘judged as having no ERM or classified as unassessed’ combines ‘no ERM’ and ‘unassessed’ judgments for binary McNemar analysis. Unassessed judgments were more frequent with Optos^®^ than Clarus™ (median per reader: 13 vs. 6; see Table 2), contributing to its lower detection accuracy. Exact distribution within the combined category is not separated in the original analysis, but the higher unassessed rate with Optos^®^ is acknowledged.

**Table 7:** Results of 10 readers’ judges to Figure 1, Figure 2, Figure 3 and Figure 4 images.

	Reader	1	2	3	4	5	6	7	8	9	10
Figure 1	Optos^®^	Y	Y	Y	Y	Y	Y	Y	Y	Y	Y
	Clarus^TM^	Y	Y	Y	Y	Y	Y	Y	Y	Y	Y
Figure 2	Optos^®^	Y/N	N	N	Y	N	N	N	N	N	Y/N
	Clarus^TM^	Y	Y	Y	Y	Y	Y	Y	Y	Y	Y
Figure 3	Optos^®^	N	N	N	N	N	N	N	N	N	N
	Clarus^TM^	Y	Y	Y	Y	Y	Y	Y	N	Y	Y
Figure 4	Optos^®^	Y/N	Y/N	N	N	N	N	Y/N	N	N	N
	Clarus^TM^	N	N	N	N	N	N	N	N	Y/N	N

Y, yes (ERM present). N, no (ERM absent). Y/N, yes or no (unassessed).

## Data Availability

All data generated or analyzed during this study are included in this article. Further inquiries can be directed at the corresponding authors.

## Supplementary Materials

The following supporting information can be downloaded at: https://www.mdpi.com/article/10.3390/jcm15020883/s1. Supplementary Table S1. Subgroup analysis for each rater.
