# Supplementary material for: Comparison of Epiretinal Membrane Detection Rates Between Optos® and Clarus™ Ultra-Widefield Fundus Imaging Systems
Source: J Clin Med. 2026 Jan 21;15(2):883. doi: 10.3390/jcm15020883 (PMC12841922; doi:10.3390/jcm15020883)
Supplement: Supplementary file 1 [file jcm-15-00883-s001.zip › jcm-3987324-supplementary.pdf]

Supplementary Table S1. Subgroup analysis for each rater.

Rate (%)	Grader experience	Optos®	Clarus™
Sensitivity	1 year (n=4)	12 (4-15)	49 (45-53)
	At least 2 years (n=6)	27 (9-47)	50 (42-70)
False positives (wrongly judged as present)	1year (n=4)	0 (0-1)	3 (2-3)
	At least 2 years (n=6)	2 (0-5)	5 (1-11)
Specificity	1 year (n=4)	91 (64-97)	94 (88-96)
	At least 2 years (n=6)	85 (48-94)	89 (87-92)
False negatives (wrongly judged as absent)	1 year (n=4)	68 (43-85)	45 (30-49)
	At least 2 years (n=6)	50 (23-77)	38 (28-49)
Correct judges (rightly judged as present or absent)	1 year (n=4)	80 (57-87)	88 (84-90)

	At least 2 years (n=6)	76 (44-88)	84 (82-86)
Wrong judges (wrongly judged as present or absent)	1 year (n=4)	9 (6-12)	8 (7-8)
	At least 2 years (n=6)	9 (4-12)	10 (6-13)
Unassessed	1 year (n=4)	10 (3-37)	5 (2-10)
	At least 2 years (n=6)	14 (3-52)	6 (2-13)

ERM, epiretinal membrane. Data are presented as median (range).
